# Impact of the increase in the number of community pharmacists on their geographical distribution in Japan: a retrospective survey

**DOI:** 10.1186/s40545-022-00499-9

**Published:** 2022-12-08

**Authors:** Kazuhiro Iguchi, Midori Ueyama, Hiroto Nishio, Hirofumi Tamaki, Arihiro Osanai, Yoko Ino, Kazuya Nonomura, Megumi Horibe, Toshiyuki Matsunaga, Mitsuhiro Nakamura

**Affiliations:** 1 Laboratory of Community Pharmacy, Gifu Pharmaceutical University, 1-25-4 Daigaku-Nishi, Gifu, 501-1196 Japan; 2 Mamiya Dispensing Pharmacy, 2-15 Kiyozumi-Cho, Gifu, 500-8178 Japan; 3 Department of Nursing, School of Health Science, Asahi University, 1851 Hozumi, Mizuho, Gifu, 501-0296 Japan; 4 Education Center of Green Pharmaceutical Sciences, Gifu Pharmaceutical University, 5-6-1 Mitahora-Higashi, Gifu, 502-8585 Japan; 5 Laboratory of Drug Informatics, Gifu Pharmaceutical University, 1-25-4 Daigaku-Nishi, Gifu, 501-1196 Japan

**Keywords:** Community pharmacists, Maldistribution, Area of inhabitable land, Population, Pharmacist density

## Abstract

**Background:**

Appropriate distribution of health care resources is required to adjust regional disparities in the quality of health care. Besides, the number of community pharmacists in Japan has increased recently, but the impact of this increase on the distribution of community pharmacists is unknown. Thus, we aimed at investigating the effect of the increase in the number of community pharmacists on the distribution per population and per area of inhabitable land.

**Methods:**

Data from 2008 to 2018 were used. Equity among municipalities in the number of community pharmacists per population and per area of inhabitable land was assessed using the Gini coefficient. A mosaic plot was used to demonstrate the relationship between the population density and increase in the number of community pharmacists per municipality.

**Results:**

The number of community pharmacists increased by approximately 1.3-fold from 2008 to 2018 in Japan. The Gini coefficient per population decreased gradually, whereas that per area increased slightly, with no change in distribution per area of inhabitable land. The number of community pharmacists per population increased regardless of the population density, but this increase per area was smaller for lower population density groups and larger for higher population density groups.

**Conclusion:**

The increase in the number of community pharmacists has improved the distribution of community pharmacists per population, but not that per area of inhabitable land. The maldistribution of community pharmacists per area implies an imbalance in the distance between pharmacies and residents. Thus, there is need for measures to improve the distribution of community pharmacists.

## Background

The substantial history of the separation of dispensing and prescribing drugs in Japan is said to have begun in 1974. Subsequently, since the 1990s, the number of outpatient prescriptions increased markedly with the full-scale progress of the separation [1, 2]. This was accompanied by an increase in the number of community pharmacies, and the shortage of pharmacists became a serious concern, especially in rural areas [3]. Meanwhile, since 2003, a number of pharmacy schools have been established, increasing from 46 in 2002 to 75 in 2019 [4, 5]. Enrollment in pharmacy schools increased approximately 1.4-fold from 8200 in 2002 to 11,487 in 2019 [5]. This has led to an increase in the number of community pharmacists [6–8].

Japan's population peaked in 2004 and has been declining ever since, although the aging rate continues to rise. The percentage of the population aged ≥ 75 years is estimated to reach 19.2% by 2030 and 25.2% by 2065 [9]. In addition, approximately 30% of the population aged 75 and over is certified as requiring support or nursing care [10]. Thus, there is no doubt that the burden of caregiving and the need for home care support will increase in the future. Considering this current situation, the establishment of a “community-based integrated care system”, is being promoted to enable residents to continue living in their familiar communities [9, 11, 12]. The “community-based integrated care system” aims to provide medical care, nursing care, and daily life support in an integrated manner, and is established through the cooperation of many human resources, including healthcare professionals, nursing care professionals, government officials, and local residents [9, 11, 12]. As a component of this system, community pharmacists are expected to promote residents’ health, specifically by offering home healthcare services and acting as a family pharmacist in a health support pharmacy [13–16]. That is, the appropriate placement of community pharmacists is essential for establishing a foundation for a community symbiotic society in a super-aging society.

However, as of 2010, the distribution of community pharmacists in Japan was concentrated only in urban areas [6]. Our previous study also showed that as of 2014, the distribution of community pharmacists was skewed, with several towns and villages having no community pharmacists, especially in rural areas [17]. Here, it is possible that the increase in the number of community pharmacists due to the establishment of several new pharmacy schools improved the distribution of pharmacists in areas with few pharmacists, but it is unclear what the actual situation was. This study aimed to ascertain whether the regional distribution of pharmacists improved because of the country’s growing pharmacist population. In addition to the bias in the number of regional pharmacists per population, this study focused on the bias per area to consider the distance between pharmacies and patients' homes.

## Methods

### Dataset

The number of community pharmacists per year was obtained from “Survey of Physicians, Dentists and Pharmacists (Ministry of Health, Labour and Welfare)”. The data of the population and the area of inhabitable land in each municipality (city/ward/town/village) were obtained from “Population, its dynamics and number of households based on the Basic Resident Register (Ministry of Internal Affairs and Communications)” and “Statistical Observations of Municipalities (Ministry of Internal Affairs and Communications)”, respectively. We obtained and used data from 2-year intervals from 2008 to 2018. All data were downloaded from the e-Stat (a portal site for Japanese Government Statistics) of the National Statistics Center [18] in December 2021. Data for each year were compiled based on 2018 municipal boundaries and the number of municipalities was fixed at 1741. Data on the area of inhabitable land in the municipalities of Kofu City, Yamanashi Prefecture and Fujikawaguchiko Town, Yamanashi Prefecture were missing until 2011 after the 2006 municipal merger. Therefore, the 2011 data were used for the area of inhabitable land in 2008 and 2010.

### Gini coefficient

We used the Gini coefficient as the indicator of equity among municipalities. The Gini coefficient is a measure of inequality in income distribution and is used in assessing the maldistribution of health care resources. The coefficient for the numbers of community pharmacists was calculated based on the population size or the area of inhabitable land per municipality. The Gini coefficient of the number of community pharmacists per population was obtained using a Lorenz curve of the cumulative relative frequency of the population versus the cumulative relative frequency of the number of community pharmacists when the municipalities were arranged in increasing order of the number of community pharmacists per 100,000 persons. Similarly, the Gini coefficient of the number of community pharmacists per area of inhabitable land was estimated using a Lorenz curve of the cumulative relative frequency of the area of inhabitable land versus the cumulative relative frequency of the number of community pharmacists when the municipalities were arranged in increasing order of the number of community pharmacists per area. The coefficient varies between 0 (complete equity) and 1 (complete inequity).

### Mosaic plot

We constructed mosaic plots of the relationship between the population density of municipalities and proportion of municipalities where the increase in the number of community pharmacists deviated from the median. The area of the rectangle in this plot indicates the ratio of the analysis target.

## Results

**Fig. 1:**
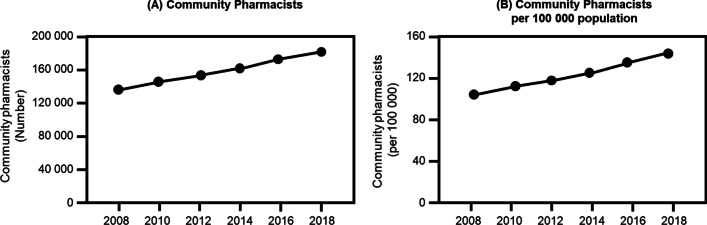
Trends in the number of “community pharmacists” and “community pharmacists per 100 000 population”

**Fig. 2:**
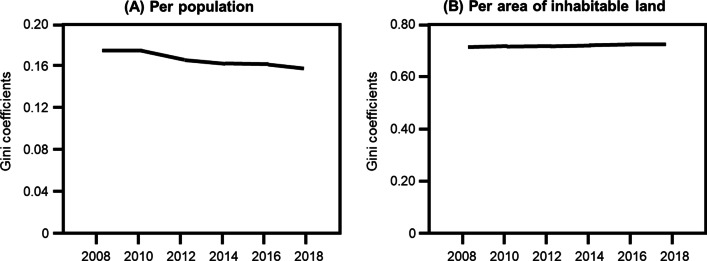
Trends in Gini coefficients. Gini coefficients of the number of community pharmacists per population (**A**) and per the area of inhabitable land (**B**) from 2008 to 2018

The number of community pharmacists in Japan increased from 135,716 in 2008 to 180,415 in 2018 (Fig. 1A). Similarly, a continuous increase in the number of community pharmacists per 100,000 population from 106.3 in 2008 to 142.7 in 2018 in Japan was observed (Fig. 1B). Figure 2 shows the Gini coefficients from 2008 to 2018, based on municipality’s population (A) and area of inhabitable land (B), respectively. The Gini coefficients per population decreased gradually from 0.171 in 2008 to 0.156 in 2018. Conversely, the Gini coefficients per area increased slightly from 0.702 in 2008 to 0.719 in 2018.

**Fig. 3:**
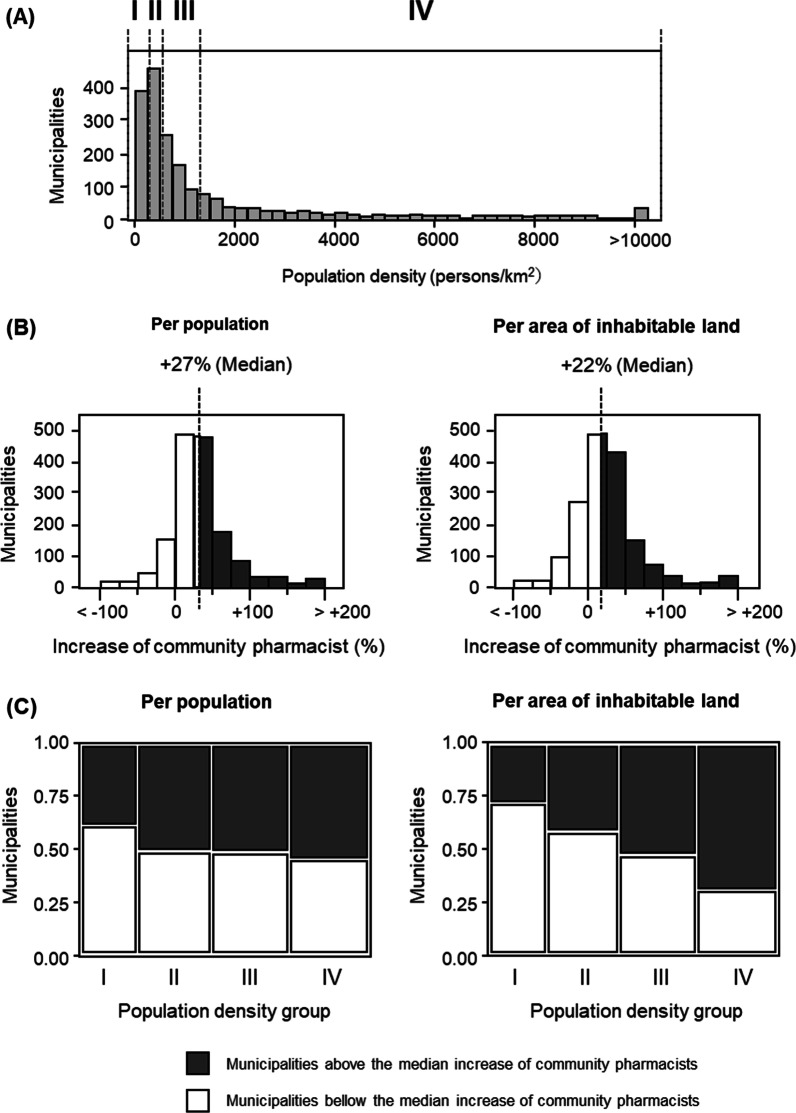
Change in the number of community pharmacists to population or area of inhabitable land per municipality. **A** Histogram of population density distribution of municipalities. The number of municipalities is divided equal into four groups, and the groups are labeled I, II, III, and IV in increasing order of population density. **B** Histogram of the change in the distribution of the number of community pharmacists to population or area to number of community pharmacists in the municipality from 2008–2018. The dotted line represents the median value. **C** Mosaic plots of 2008–2018 change in the number of community pharmacists vs. population or area vs. number of community pharmacists for municipalities

To examine the relationship between municipal population density and increase in the number of community pharmacists, all municipalities were divided into quartiles of equal size according to population density (Fig. 3A); and the average number of community pharmacists per population and per area of inhabitable land in each quartile was calculated per year (Table 1). The average number of community pharmacists per population increased by approximately 1.3 times in all population density groups in 2018 compared to that in 2008. However, the average number of community pharmacists per area experienced a smaller increase in the lower population density groups, and a larger increase in the higher population density groups: from Class I to Class IV (1.12- to 1.36-times).

**Table 1:** Trends in the number of community pharmacists per population (A) and per area of inhabitable land (B) from 2008 to 2018

	*N*	2008	2010	2012	2014	2016	2018	Fold increase (2018/2008)
(A) Number of pharmacists per 100,000 population								
All	1741	73.7	78.5	81.9	86.0	91.7	96.3	1.31
I	435	42.2	45.1	47.5	49.6	53.1	55.6	1.32
II	435	64.3	68.2	70.5	74.7	78.9	83.6	1.30
III	436	83.8	89.2	93.2	97.6	103.4	108.7	1.30
IV	435	104.7	111.6	116.3	121.9	131.5	137.5	1.31
(B) Number of pharmacists per area of inhabitable land (km^2^)								
All	1741	1.49	1.59	1.67	1.76	1.89	1.98	1.34
I	435	0.07	0.07	0.07	0.07	0.07	0.08	1.12
II	435	0.26	0.27	0.27	0.29	0.29	0.30	1.16
III	436	0.71	0.74	0.77	0.80	0.84	0.87	1.23
IV	435	4.91	5.28	5.57	5.87	6.35	6.69	1.36

Subsequently, for each municipality, we compared the increase factor of the number of community pharmacists per population and per area of inhabitable land in 2018 to those in 2008. Municipalities (177) with zero pharmacists in 2008 were excluded from the analysis (because the denominator would be zero), thus, reducing the total number of eligible municipalities to 1564. The median change in the number of community pharmacists per population and per area of inhabitable land was + 27% and + 22%, respectively (Fig. 3B). Mosaic plots were then constructed to examine the distribution of municipalities with increases different from the median increase in each of the four groups of municipalities according to population density. The population density groups exhibited an obscure relationship with the ratio of municipalities with high increase of community pharmacists per population (Fig. 3C), however, there was an evident correlation between the population density group and the ratio of municipalities with high increase of community pharmacists per area.

## Discussion

In this study, we examined the impact of the increase in the number of community pharmacists on their regional distribution. The Gini coefficients for community pharmacists per population showed a decreasing trend over time, whereas those per area of inhabitable land showed a slightly increasing trend. Thus, the increase in the number of community pharmacists has improved the distribution of the number of community pharmacists per population but not per area.

Japan's recent ageing population has necessitated a regional system that can provide the appropriate home health care [9, 11, 12]. Thus, services provided by community pharmacists are becoming more relevant. The maldistribution of community pharmacists per area implies the increase of travel distances from the pharmacy to homes of patients, causing an overburden on the community pharmacist to provide services. Moreover, accessibility to medical resources, including community pharmacies, is an important factor for residents to live in a community. Therefore, optimization of the geographic arrangement of limited medical resources is of utmost relevance. Besides, similar issues with geographic maldistribution of health care resources per area have been reported in other countries [19–23]. For example, the distribution of physicians, nurses, and hospital beds in Mongolia is less skewed relative to population, but extremely skewed relative to inhabitable area [23]. This study points out the need for measures that can adjust for geographic imbalances, not only by simply increasing the number of medical resources, but also by influencing the lifestyles of the population [23].

The increase in the number of pharmacists in Japan has improved the distribution of pharmacists per population. This can be attributed to a phenomenon similar to the market principle: the increase in the number of pharmacies in urban areas peaked, which were distributed to rural areas with a shortage of community pharmacists. However, the increase in the number of physicians in Japan has not eliminated their maldistribution per population [24, 25]. This is probably because physicians can maintain profitability by inducing demand even in large cities that appear to have attained saturation [24]. Additionally, the distribution of physicians in the U.S. followed the income distribution of residents [24]. Interestingly, these reports contrasted the results of this study. These differences may be due to the fact that most community pharmacists in Japan receive their income from dispensing prescriptions, making it difficult for pharmacists themselves to create demand for medical care; and the universal health insurance system in Japan makes it difficult to follow economic benefits.

The number of municipalities without a community pharmacist has decreased from 177 in 2008 to 149 in 2018 (data not shown). This indicates an improvement in distribution of community pharmacists per population. In general, it is assumed that municipalities without a community pharmacist have a relatively small population and a large area. Even if community pharmacists were assigned, the improvement in the number of community pharmacists per area would be limited. This makes it unrealistic to expect such an area to have a large number of community pharmacists per area. In areas where there is a shortage of pharmacists, adequate medical care may not be provided to the local population, and pharmacists working in these areas may be overworked. A possible solution to eliminate regional disparity in medical resources per inhabitable area is to use online technology, such as telepharmacy, which is expected to develop more in the future and reduce geographic constraints [26–29]. Besides, the efficiency of pharmacy management through the mechanization of pharmacy operations may create time for pharmacists. In addition, the introduction of a pharmacy technician system, which performs some of the dispensing tasks, will create more room for pharmacy management [30, 31]. Although a pharmacy technician system has not been introduced in Japan, in April 2019, the Ministry of Health, Labour and Welfare has just mentioned pharmacy services that can be performed by non-pharmacist staff [32]. These may help rectify the geographic imbalance of community pharmacists.

The limitations of this study are as follows. First, we assumed that the number of community pharmacists per area of inhabitable land correlates with the distance and travel time between pharmacies and residents. Data aggregated by the administrative division, as in this study, includes the possibility of discrepancies with the actual scenario, such as the case of pharmacies located near the boundaries of municipalities. Evaluating accessibility to medical resources based on road and public transportation information can improve the estimation of distribution. Although the present report is an important first step in evaluating accessibility, future studies should consider facility layout evaluation using geographic information systems. Second, it is necessary to consider the distribution of pharmacies in addition to community pharmacists in the region, but the present study referred only to the number of community pharmacists. Since the number of community pharmacists in this study is based on the number of pharmacists in the municipality where the pharmacists work, pharmacies can be considered to be located in that municipality. Thus, the analysis of the distribution of community pharmacies, in addition to the distribution of pharmacists, is recommended as it allows for a deeper evaluation of the community health care system.

## Conclusions

The recent increase of community pharmacists in Japan has improved their distribution per population but not area. Thus, the increase in community pharmacists was evenly distributed according to population, not area. Geographic imbalance must be considered when designing policies for effective use of limited human resources. More aggressive and multifaceted measures, beyond increasing the number of pharmacists, are required to correct the uneven access to pharmacists among regions.

## Data Availability

Data used in this study are available from the following links: https://www.e-stat.go.jp/stat-search/files?page=1&toukei=00450026&tstat=000001030962,https://www.e-stat.go.jp/stat-search/files?page=1&toukei=00450026&tstat=000001135683; https://www.e-stat.go.jp/stat-search/files?page=1&layout=datalist&toukei=00200241&tstat=000001039591&cycle=7&tclass1=000001039601&tclass2val=0; https://www.e-stat.go.jp/regional-statistics/ssdsview.
